# PPAR*δ*, a Potential Therapeutic Target for Heart Disease

**DOI:** 10.32527/2018/101375

**Published:** 2018-10-30

**Authors:** Qinglin Yang, Qinqiang Long

**Affiliations:** 1 Cardiovascular Center of Excellence, LSU Healther Science Center, 533 Bolivar St, New Orleans, LA 70112, USA; 2 Division of Cardiology, Department of Internal Medicine, Tongji Hospital, Tongji Medical College, Huazhong University of Science and Technology, 1095 Jiefang Ave, Wuhan, 430030, China

## Abstract

The nuclear receptor peroxisome proliferator-activated receptor *δ* (PPAR*δ*) can transcriptionally regulate target genes. PPAR*δ* exerts essential regulatory functions in the heart, which requires constant energy supply. PPAR*δ* plays a key role in energy metabolism, controlling not only fatty acid (FA) and glucose oxidation, but also redox homeostasis, mitochondrial biogenesis, inflammation, and cardiomyocyte proliferation. PPAR*δ* signaling is impaired in the heart under various pathological conditions, such as pathological cardiac hypertrophy, myocardial ischemia/reperfusion, doxorubicin cardiotoxicity and diabetic cardiomyopathy. PPAR*δ* deficiency in the heart leads to cardiac dysfunction, myocardial lipid accumulation, cardiac hypertrophy/remodeling and heart failure. This article provides an up-today overview of this research area and discusses the role of PPAR*δ* in the heart in light of the complex mechanisms of its transcriptional regulation and its potential as a translatable therapeutic target for the treatment of cardiac disorders.

## 1. Introduction

Peroxisome proliferator-activated receptors (PPAR*α*, -*γ* and -*δ*) belong to a nuclear receptor transcription factor superfamily that regulates metabolic transcription. Ligands of PPAR*α* and -*γ* are clinically effective in treating dyslipidemia and insulin resistance, although adverse risks have remained a concern (see review [1]). PPAR*δ* (NR1C2, encoded by the Ppard gene, also known as PPAR*β*) was first cloned in the early 90’s of the 20th century [2, 3]. No FDA approved drug targeting PPAR*δ* is available so far notwithstanding early-stage clinical trials on the use of PPAR*δ* agonists (e.g., KD-3010 and MBX-8025) to treat atherosclerosis, metabolic disorders and liver injury [4–6]. Early-stage clinical trials of selective PPAR*δ* agonists Seladelpar (MBX-8025) in patients with primary biliary cholangitis was terminated due to grade 3 increases in serum transaminase levels [7]. Therefore, a PPAR*δ*-specific therapeutic strategy remains in perspective with improved selectivity and minimal off-target effects.

As the most energy demanding organ, energy metabolism is essential for the normal function of hearts. A normal adult heart predominantly relies on the energy from fatty acid oxidation (FAO). PPARs play essential roles in transcriptional regulation of many aspects of cellular metabolism, especially FAO. All three PPAR subtypes are expressed in heart cells (cardiomyocytes) [8–11]. PPAR*α* controls the transcriptional expression of key enzymes that are involved in FA uptake and oxidation, triglyceride synthesis, mitochondrial respiration uncoupling and glucose metabolism [10, 12, 13]. The role of PPAR*γ* in the heart has not been extensively studied due to its relatively low abundance. However, several studies on cardiomyocyte-restricted PPAR*γ* mice demonstrate its crucial role in cardiac physiology, probably via its role in transcriptionally regulating myocardial FA metabolism and anti-oxidant defense [14–17]. The cardiac function of PPAR*δ* been explored until subtype-specific synthetic ligands for PPAR*δ* became available in the early 2,000’s. In the heart, PPAR*δ* is prominently expressed in cardiomyocytes and activation of PPAR*δ* upregulates FAO transcripts as in cultured cardiomyocytes [18, 19]. Global PPAR*δ* KO in mice is embryonically lethal due to placenta defects [20]. Survival PPAR*δ* KO mice show an extremely lean phenotype [20]. Considering that potential compensation may conceal potential phenotypic manifestation, conditional PPAR*δ* KO mouse lines were developed [20–29]. Inborn cardiomyocyte-restricted PPAR*δ* deletion leads to cardiomyopathy and heart failure with impaired myocardial FAO [30]. Therefore, it becomes clear that all PPAR subtypes play roles in transcriptional regulation of myocardial energy and lipid homeostasis, but with differences in their cardiac expression levels and yet to defined differential regulation on various aspects of energy metabolism. Further studies on the potential distinctive roles of each PPAR subtype in the heart should provide new therapeutic targets for treating heart disease. In this review, we focus on summarizing the biological function and clinical implications of PPAR*δ* in the heart.

## 2. PPARδ Plays a Crucial Role in Cardiac Pathophysiology

*The optimal coupling of metabolism and function is essential for the normal structure/function of the heart. Cardiac metabolism is a key player in the development of cardiac pathophysiology as FAO provides a majority of energy for a normal heart to properly function* [31, 32]. Metabolic adaptation becomes essential to meet the energy needs of the pathological heart ensue with various stimuli, such as mechanical overload, ischemia/reperfusion, aging, diabetes and drug cytotoxicity. Others and our studies documented that PPAR*δ* is a key transcription factor that regulates almost all steps of metabolism (see review [9, 33], hence PPAR*δ* can play a crucial role in determining cardiac pathophysiology.

Under various cardiac hypertrophy conditions, most studies show cardiac PPAR*δ* is decreased [34, 35] as those of PPAR*α* [12, 13]. PPAR*δ* is reduced in hypertrophic cardiomyocytes induced by angiotensin II [34] and hypertrophic hearts resulted from transverse aortic constriction (TAC)-induced left ventricular pressure overload [35]. The impaired cardiac PPAR*δ* expression could be reversed by anti-hypertrophic treatment [34, 36]. Studies using Western blots also showed protein contents of cardiac PPAR*δ* are downregulated in doxorubicin-cardiac cytotoxicity [37] and STZ-induced diabetic hearts [38]. In contrast to the above investigations, another study using Western blot showed that cardiac PPAR*δ* is upregulated in the heart with infarction in rats [39]. The reason for these different findings of PPAR*δ* in pathological hearts could be related to the specific pathological condition, the animal species/strain, and the technical issues of antibody specificity.

While changes of PPAR*δ* expression in the pathological heart need further validation, the essential role of cardiac PPAR*δ* is well established. Cardiomyocte-restricted PPAR*δ* deletion was achieved by *breeding* the floxed mice with a mouse line with cardiomyocyte-restricted expression of Cre recombinase (α-Myosin heavy chain-Cre). Inborn cardiomyocyte-restricted PPAR*δ* deletion impairs myocardial FAO and leads to cardiomyopathy and heart failure [30]. To further gain insights into the function of PPAR*δ* in the adult heart, we generated mice with Tamoxifen-induced cardiomyocyte-restricted PPAR*δ* deletion by crossing the floxed PPAR*δ* mice with a mouse line carrying α-Myosin heavy chain-Mer-Cre-mer. Adult mice with cardiomyocyte-restricted PPAR*δ* deletion consistently show cardiac dysfunction, cardiac hyper-trophy and remodeling [40]. On the other hand, studies using *gain-of-function* approaches also mostly support a protective effect of PPAR*δ* in the heart. Transgenic mice with cardiomyocyte-restricted expression of a constitutively active form of PPAR*δ* show no overt cardiac phenotype, yet these mice become resistant to pressure overload-induced cardiac dysfunction [41].

*Since the role and activity of PPARδ is not entirely dependent on its transcript or protein abundance but also on the molecular regulation that PPARδ exerts as gene inducer or repressor, studying the effects of pharmacological activators is therefore complementary to abundance studies*. PPAR*δ* selective ligands reduce hypertrophic response induced by phenylephrine in cultured rat neonatal cardiomyocytes and rat embryonic myocytes (H9C2) [42]. PPAR*δ* activation improves angiotensin II-induced cardiac hypertrophy *in vitro* [43]. However, a later study on cultured rat adult cardiomyocytes shows upregulation of PPAR*δ* activity and FAO gene expression with minimal effect on cell growth [43]. *It remains unclear whether the discrepancy is related to the differentiation states of cardiomyocytes or the culture conditions used in the studies*. In vivo pharmacological studies in the literature provide further support. An early study on rats shows the PPAR*δ* agonist GW0742 protects against right heart hypertrophy from post-myocardial infarction [44]. Pharmacological studies show that baicalin and Moringa oleifera Seeds, both extracted from plants, protect the heart from TAC-induced pressure overload or hypertension from the development of hypertrophy via enhancing cardiac PPAR*δ* expression [35, 45]. The cardiac protective effect of PPAR*δ* is further supported by studies in animal models with myocardial ischemia/reperfusion (IR) injury. Activation of PPAR*δ* attenuates myocardial IR injury in rats [39] and mice [46]. Our results from a study on mice with cardiomyocyte-restricted transgenic expression of the constitutively active PPAR*δ* supports the protective role of PPAR*δ* activation. Mice with PPAR*δ* activation showed improved cardiac function, reduced infarct size, and fibrotic remodeling [47]. *Treatment with GW0742* protects the heart in a PPAR*δ*- dependent manner [47]. It is interesting that another study on the effect of permanent myocar-dial infarct in rats shows functional effects of PPAR*δ* agonist (GW0742) treatment, but with augmented cardiac fibrosis with no functional consequece [48]. However, a control group to determine the basal effect of PPAR*δ* agonist (GW0742) was not included in this study, missing the opportunity to determine if GW0742 treatment alone could induce cardiac fibrosis. It is noted that the study assigned hearts to different groups based on the degree of infarction determined by echocardiographic images were used for all experimental procedures. The overtly biased experimental design may oblivion correct interpretation of the outcomes. Further studies with careful experimental design should help clarify the conclusions.

The cardiac protective role of PPAR*δ* activation has been validated further in other advert states. Cardiac cytotoxicity is a main clinical dilemma in the use of effective anti-cancer drugs. Evidence has emerged that activation of PPAR*δ* protects against doxorubicin-induced cardiotoxicity. A PPAR*δ* activator, L-165041, inhibits senescence in neonatal rat cardiomyocytes and H9C2 cells via interacting with Bcl6 to reduce apoptosis [49]. On the other hand, PPAR*δ* ligand GW0742 exerts a protective role in neonatal rat cardiomyocyte treated with Doxorubicin by sustaining intracellular calcium concentration [50]. Baicalein, a natural PPAR*δ* ligand, protects against doxorubicin-induced cardiotoxicity by attenuation of mitochondrial oxidant injury and JNK activation [51].

Diabetes (type 1 and type 2) and obesity have a strong link to cardiac dysfunction, cardiac hypertrophy and heart failure (see review [52–54]). Normalizing cardiac PPAR*δ* protein improves cardiac fibrosis in rats with streptozocin (STZ)-induced diabetes [37]. Induction of cardiac angiopoietin Like 4 (Angptl4) via PPAR*δ* activation in the heart is essential in protecting against FA-induced oxidative stress [55], a key mechanism behind diabetic cardiomyopathy. Several reports indicate that restoring PPAR*δ* protein expression in diabetic hearts from rats induced by STZ by various pharmacological compounds, such as histone deacetylase (HDAC) inhibitor [56], ginseng [9, 57] and ramipril [58]. However, many of these preliminary findings need further validation. Further studies are required to assess the PPAR*δ* expression in the heart in transcript level, target gene expression and subtype-specific activity to confirm the observations.

**Figure 1:**
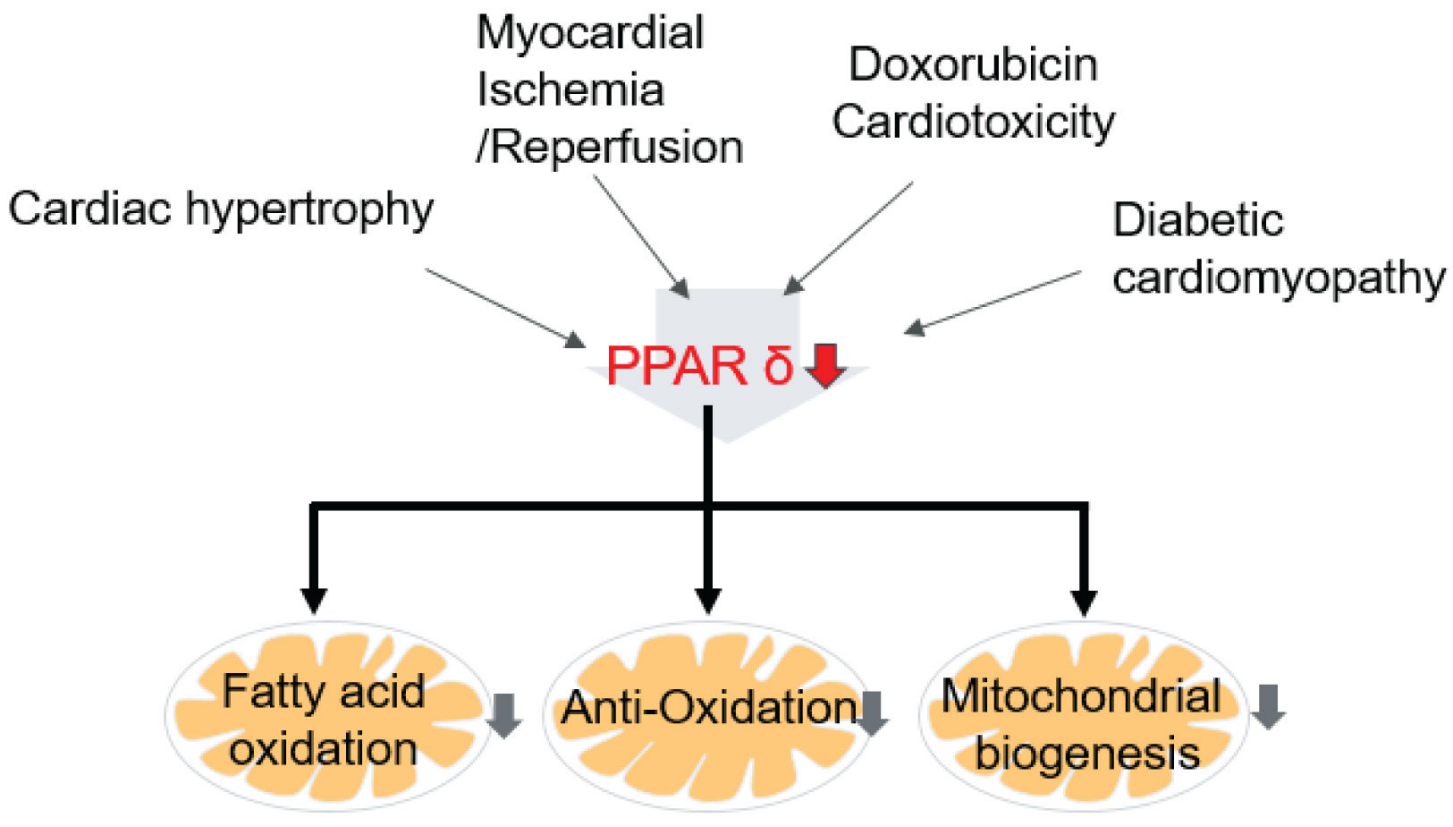
PPAR*δ* plays a crucial role in cardiac pathophysiology: PPAR*δ* signaling is impaired in the heart under various pathological conditions, such as pathological cardiac hypertrophy, myocardial ischemia/reperfusion, doxorubicin cardiotoxicity and diabetic cardiomyopathy. PPAR*δ* deficiency in the heart leads to cardiac dysfunction, myocardial lipid accumulation, cardiac hypertrophy/remodeling and heart failure.

In summary, It is apparent that most of the current studies illustrate that cardiac PPAR*δ* may be repressed in cardiac disorders and PPAR*δ* re-activation in the heart is protective (Figure 1), whereas PPAR*α* re-activation in the heart could be detrimental [59, 60] or beneficial [61–64], possibly depending on the degree of activation.

## 3. Molecular and Biochemical Mechanisms Underlying the Biological Action of PPARδ in the Heart

### 3.1. PPARδ is a key transcriptional regulator of FA utilization in the heart

As a ligand-activated nuclear receptor and transcription factor, PPAR*δ* regulates transcription in many tissues (see review [9, 65, 66]). Being ubiquitously expressed in the body, PPAR*δ* is expressed preferentially in cardiomyocytes among other cell types in the heart [19]. In rat neonatal cardiomyocytes (RNCM), PPAR*δ* regulates transcript expression of key genes involved in FA metabolism [19]. PPAR*δ*-selective ligand and PPAR*δ* overexpression in cultured RNCM promotes FA metabolic gene expression and the rate of FAO in a classic ligand binding dependent mechanism [19]. We and other further confirmed that PPAR*δ* regulates the transcriptome of FA utilization in the heart (see reviews [9, 67, 68]). PPAR*δ* is a key determinant of FA uptake in cardiomyocytes by upregulating FA uptake genes, such as FA transport protein (FATP) and FA binding protein (FABP) [18, 19, 30, 40, 41, 69, 70]. Moreover, PPAR*δ* ligand treatment not only upregulates key mitochondria-specific *β*-oxidation genes, such as Long-chain acyl-CoA synthetase (LACS), carnitine palmitoyltransferase very-long-chain acyl-CoA dehydrogenase (VLCAD), mitochondrial 3-ketoacyl-CoA thiolase, CPT-A, uncoupling protein 2 (UCP2), UCP3, pyruvate dehydrogenase kinase 4 (PDK4), medium chain acyl CoA dehydrogenase (MCAD), long chain acyl CoA dehydrogenase (LCAD) and malonyl CoA decarboxylase (MCD), but also peroxisome-specific *β*-oxidation genes, such as acyl-CoA (ACO), and peroxisomal 3-ketoacyl-CoA thiolase [18, 19, 30, 40, 41, 69]. Further studies on cardiac-specific PPAR*δ* mice provide definitive evidence supporting the role of PPAR*δ* in maintains constitutive FA metabolism in the heart [19]. In addition to depressed FAO, cardiomyocyte-restricted PPAR*δ* knockout mice develop severe phenotypic changes, such as cardiac dysfunction, myocardial lipid accumulation and progressive heart failure. The dominant expression of PPAR*δ* in cardiomyocytes of the heart explains at least in part the reason for mice with cardiomyocyte-restricted PPAR*δ* knockout exhibit a devastating pathological consequence [19, 30]. Thus PPAR*δ* may play a crucial role in metabolic homeostasis in the heart by maintaining intracellular FA content and a high-level FA metabolism observed in normal adult hearts.

Crosstalk or compensatory expression between PPAR*δ* and the other PPAR isoforms has been identified in the heart [69]. Nonetheless, pharmacological or genetically PPAR*δ* over- expression or activation did not modify the expression of PPAR*α* and PPAR*γ* in the heart in both physiological and pathological states [41, 69]. PPAR*α* and PPAR*δ* both regulate FA utilization in cardiomyocytes in an interdependent manner. Deletion of either of them from cardiomyocytes does not affect other PPARs’ effects on activating FA utilization gene expression [19, 30]. Interestingly, cardiomyocyte-restricted PPAR*δ* knockout, but not the conventional PPAR*α* knockout mice, exhibit neutral lipid accumulation in myocardium at basal state [19, 71], probably due to the mismatch of PPAR*δ* activated FAO and lipid uptake. Until further investigation on a cardiomyocyte-restricted PPAR*α* KO mouse line, it is impossible to draw a definitive conclusion on the differentiated roles of these two important transcription factors in the heart. Interestingly, a study on a mouse line with cardiac-specific overexpression of a wild-type PPAR*δ* results in increased cardiac glucose uptake and oxidation in concomitant upregulation of glucose transporter type 4 (GLUT4) and phosphofructokinase (PFK) genes [46]. However, it remains unknown if these two genes are direct targets of PPAR*δ*. A recent study demonstrates that PPAR*δ* improves exercise endurance by reducing glucose and increasing fat metabolism in skeletal muscle [26]. Therefore, it is possible that the effects of PPAR*δ* on cardiac glucose metabolism may be indirect. Further studies will be warranted to clarify the direct effect of PPAR*δ* on myocardial glucose metabolism.

### 3.2. PPARδ regulates mitochondrial antioxidant defense

The normal function and structure of the heart rely heavily on the efficiency of energy supply, which is dependent on the redox homeostasis of mitochondria, the power plant of cells. Mitochondrial redox dysfunction will result in energy deficiency and excessive production of reactive oxygen species (ROS) in cardiomyocytes, impairing cardiac function and structure. Therefore, a better understanding of the mitochondrial redox regulation in the heart may yield novel therapeutic strategies for treating cardiac disorders.

In adult mouse hearts with induced PPAR*δ* knockout, both mitochondrial biogenesis and cardiac antioxidant defense are impaired [40]. Specifically, PPAR*δ* knockout from adult heart leads to oxidative damages with an impaired cardiac expression of superoxide dismutase 1 (SOD1) and SOD2 [40]. PPARα null mice with additional PPAR*δ* knockout from the heart showed similar results [69]. Both the transcript and protein expression of SOD1 and SOD2 was repressed in PPAR*δ*, but not PPARα deficient hearts [69]. No endogenous antioxidants are affected at the basal condition in the PPARα KO heart, supporting a notion that differential regulation of antioxidant expression in the heart may be the key factor contributing to the phenotypic changes in PPAR*δ* deficient hearts. The effects of PPAR*δ* activation in regulating antioxidant defense in the heart have also been validated in mouse models with cardiomyocyte-restricted overexpression of a constitutively active PPAR*δ*. With an enhanced antioxidant defense, the PPAR*δ* activating mice showed less oxidative injuries in the heart and stronger cardiac performance even under a left ventricular pressure overload condition [41]. Another study showed that activation of PPAR*δ* protects cardiomyocytes from oxidative stress-induced apoptosis by suppressing generation of reactive oxygen/nitrogen species and expression of matrix metal-loproteinases [72]. The impaired expression of antioxidants should contribute to the cardiac dysfunction in the PPAR*δ* deficient heart [30] and activation of PPAR*δ* protects the heart at least partly via upregulating antioxidant levels in the heart under pressure overload condition [40, 41]. Other than the direct transcriptional regulation, the effect of PPAR*δ* in myocardial lipid metabolism and glucose utilization [46] at various metabolic states in different pathological development stages may contribute to the antioxidant defense and myocardial protection of PPAR*δ* ligand treatment and transgenic PPAR*δ* overexpression [40, 73].

Taken together, the above investigations support mostly a protective role of PPAR*δ* in regulating transcriptional expression of multiple endogenous anti-oxidants, protecting the heart under both physiological and pathological conditions.

### 3.3. PPARδ exerts anti-inflammation effects on the heart

Chronic inflammation is involved in the pathological development of myocardial ischemia/reperfusion injury [74, 75], pathological cardiac hypertrophy and congestive heart failure [76, 77]. Although remaining invalidated in patients, anti-inflammation to specific inflammation pathways remains a potential therapeutic target for the treatment of cardiac injuries and heart failure [78, 79].

The anti-inflammatory role of PPAR*α* and PPAR*γ* [80–82], later PPAR*δ* [83], has been documented in the last decade. The anti-inflammatory effects of PPARs in the cardiovascular system have been extensively investigated, especially in their anti-inflammatory effects on macrophage foam-cell formation and atheroscleosis [84, 85]. PPAR*δ* activation blocks lipid-induced inflammatory pathways in mouse heart and human cardiac cells [86]. PPAR*δ* represses transforming necrosis factor-*α* (TNF-*α*) in cultured cardiomyocytes via regulating the nuclear factor-*κ*B (NF-*κ*B) signaling pathway. We showed that a PPAR*δ*-selective ligand, GW0742, inhibits the lipopolysaccharide (LPS)-induced TNF-*α* production in cultured cardiomyocytes [87]. All these investigations strongly support that PPAR*δ* exerts significant anti-inflammatory effects in the heart.

Several studies established a consistent mechanism for the anti-inflammatory effects of PPAR*δ* by its direct or indirect interaction with NF-*κ*B signaling [42]. An early study has shown that unliganded PPAR*δ* sequesters the transcriptional repressor protein B cell lymphoma- 6 (Bcl6) and prevents it from binding to the response elements in the promoter regions of its target genes [88]. Bcl6 is released from PPAR*δ* and inhibits inflammatory signals [88–90]. PPAR*δ* can interact with other transcription factors such as the p65 subunit of NF−κB, preventing NF−κB dependent transcription [42]. Furthermore, the roles of PPAR*δ* in curbing palmitate-induced endoplasmic reticulum stress and inducing autophagy [91] may also contribute to its anti-inflammatory effects in cardiomyocytes.

Despite the well-recognized anti-inflammatory effects of PPAR*δ* in the heart, further studies are required to translate these findings into clinical treatment of heart disease. The main issue is still related to the potential side effects of the existed PPAR*δ*-selective ligands. On the other hand, the continuous discovery of naturally occurring ligands that could at least partially activating PPAR*δ* may help identify compounds that have less side effect yet process PPAR*δ* activation effects to achieve the therapeutic goal.

### 3.4. Potential adverse effects of PPAR*δ* in the heart

Although most investigations support the beneficial effects of PPAR*δ* on the heart, potentially detrimental effects of PPAR*δ* have been reported. In cultured H9C2 cells, a cell line of myoblasts from rat embryonic hearts with few features of cardiomyocytes, pharmacological blocking of PPAR*δ* signaling mitigates the cytotoxicity of docosahexaenoic acid (DHA), a key compound found in fish oil [92]. However, further *in vivo* studies are essential to validate this *in vitro* finding. Another study shows that PPAR*δ* ligands (GW0742 or GW501516) treatment in mice leads to rapid development of cardiac hypertrophy and angiogenesis, presumably via direct activation of calcineurin [93]. However, we did not see any cardiac hypertrophic effects of GW0742 with the same dose and subcutaneous injection. The reason for the discrepant cardiac effects of systemic treatment of GW0742 is not clear, although we cannot rule out a transient reversible cardiac growth induced by the treatment with GW0742 in our study [94]. The same group went on to demonstrate that mice with induced vascular-specific overexpression of PPAR*δ* show rapid cardiac hypertrophy [95]. Furthermore, the vascular overexpression of PPAR*δ* does not protect against chronic ischemic injury of the heart [95]. However, this study does not clarify if systemic treatment of GW0742 triggers cardiac hypertrophy by its effects on the vasculature. Therefore, further studies on the PPAR*δ* effects by assessing different cell types in the heart is needed in evaluating the potential clinical use of PPAR*δ* ligands for the treatment of heart diseases.

**Figure 2:**
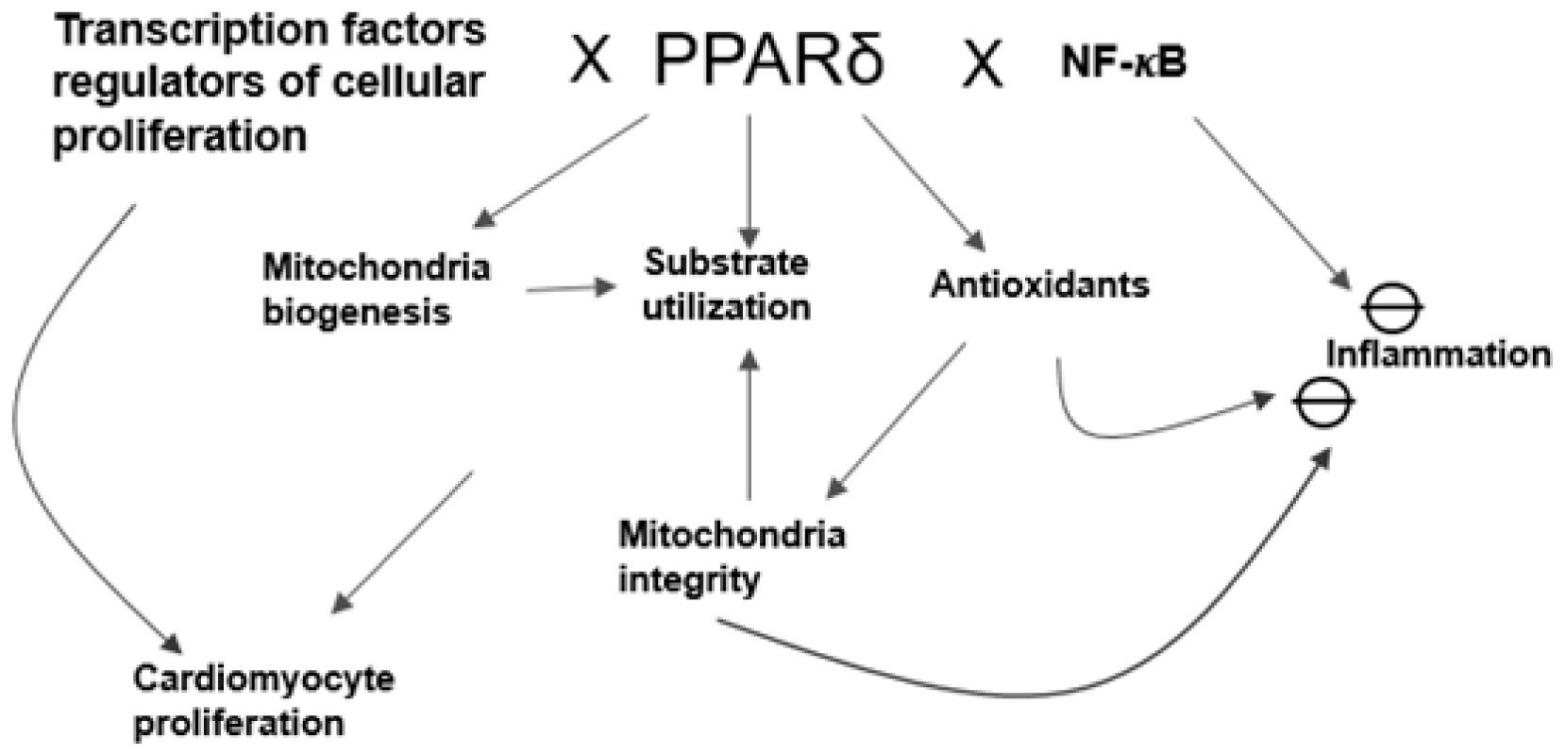
The pleiotropic and integrated effects of PPAR*δ* that affects cardiac structure and function. Investigations in the past 15 years uncovered the potential mechanisms underlying the beneficial roles of cardiac PPAR*δ* in determining cardiac pathophysiology. *In addition to direct transcription regulation of mitochondrial biogenesis, substrate utilization and antioxidant defense in the heart, PPARδ exerts anti-inflammation and cardiomyocyte proliferated effects probably via interacting with other transcription factors and cellular signaling pathways*.

In summary, the importance of PPAR*δ* in cardiac pathophysiology is multi-faceted through direct transcriptional activation and secondary modulation of other transcriptional and cellular signaling pathways. For example, the PPAR*δ* interaction with NF-kB attributes to its anti- inflammatory effects. It is also likely that the transcriptional effects of PPAR*δ* on FA utilization, mitochondrial biogenesis and antioxidant defense are further integrating with other biological effects such as inflammatory processes and cell proliferation (Figure 2). We recently uncovered that PPAR*δ* activation facilitates the cell cycle re-entry of adult cardiomyocyte [94]. Since adult cardiomyocytes are mostly terminally differentiated, enabling terminally differentiated cardiomyocyte re-entering cell cycle remains a major challenge [96–98]. Our drug screen identified carbacyclin, a PPAR*δ* activator, which induces cardiomyocyte proliferation via a PPAR*δ*/PDK1/p38/Akt/GSK3β/β-catenin-pathway. Activating PPAR*δ* signaling after myocardial infarction (MI) induces cardiomyocyte cell cycle activity and improves scarring as well as cardiac function [94]. PPAR*δ* deletion from the heart reduces the effect of PPAR*δ*-selective agonist, GW0742, on adult cardiomyocyte proliferation in mice [94]. It is likely that potential interaction of PPAR*δ* with other transcriptional factors enables cardiomyocyte proliferation in the adult heart. Future investigations should focus on how PPAR*δ* interacts with other transcription factors/signaling pathways to exert its biological functions. Further studies should also target to exploit PPAR*δ*’s many potentially beneficial effects for the treatment of common cardiac disorders.

## 4. Upstream regulation of PPARδ

Several previous studies demonstrate that cardiac PPAR*δ* is downregulated in various pathological states [34, 35, 99]. The mechanisms underlying the downregulation of cardiac PPAR*δ* expression and activity is intriguing but remains elusive.

Nuclear receptor coactivator 6 (NCOA6) is a transcription coregulator. Mouse models of NCOA6 dysfunction develop severe dilated cardiomyopathy with impaired mitochondrial function and reduced activity of PPAR*δ* [100]. It is not clear if the down-regulation of cardiac PPAR*δ* is part of the pathological consequence or the co-regulator acts through an unknown mechanism that repressing the expression of PPAR*δ*. An exact mechanism will require further investigations. PPAR*δ* is modulated by epigenetic modification. Specific microRNAs repress the expression of cardiac PPAR*δ*. For example, the hypoxia-inducible microRNA cluster miR-199a- 214 represses approximately PPAR*δ* target gene expression in the heart and impairs mitochondrial FAO [36, 70]. A recent study shows that the circadian repressors (crytochrome-1) CRY1 and CRY2 function as co-repressors for PPAR*δ*, which represses a distinct subset of PPAR*δ* target genes in skeletal muscle [101]. However, further study will be needed to determine if they exert similar PPAR*δ* repression effect in the heart.

A recent study revealed that leptin administered via intracerebroventricular enhances cardiac FAO by activating PPAR*δ* in the heart [102]. Various lipid species from the increased lipolysis by leptin down-stream signaling in the heart may serve as endogenous ligands that activate cardiac PPAR*δ* by the central leptin. PPAR*δ* can be deactivated by post-translational modification such as SUMOylation. Small ubiquitin-like modifier (SUMO)-specific proteases (SENPs) that reverse protein modification by deSUMOylation of PPAR*δ* and PPAR*γ* to enhance FA metabolism in cultured C2C12 myotubes [103]. AMP-activated protein kinase (AMPK) is well-recognized cellular energy-sensing enzyme complex [104, 105]. PPAR*δ* agonist GW501516 activates AMPK and stimulates glucose uptake in skeletal muscle [106, 107]. AMPK may interact with PPAR*δ* to exert their gene regulation function *with unknown mechanisms* [108].Given the well-established beneficial effects of AMPK activation in the heart (see review [109]) and similarity of skeletal muscle and heart muscle, it is plausible that similar AMPK activation effects of PPAR*δ* activation may also apply to the heart. However, it remains obscure how AMPK is activated by PPAR*δ*, which presumably leads to increase energy metabolism. Further investigations are warranted. PPAR*δ* can also be activated by an array of long-chain FAs and prostaglandins and other metabolites. However, the specificity and potency of these endogenous ligands appear to be relatively modest. Several selective PPAR*δ* agonists have been developed and well documented, such as GW0742, GW501516, and L165041 [110–113]. Synthetic PPAR*δ* antagonists, such as GSK-3787, are also available [114]. Several clinically used therapeutic compounds have been reported to be able to enhance the activity of PPAR*δ* in addition to their specific therapeutic targets. Ramipril is an angiotensin-converting enzyme inhibitor, used to treat high blood pressure and congestive heart failure [115]. Interestingly, Ramipril can be normalized the decreased PPAR*δ* and PPAR*γ* mRNA in animals treated with the anti-cancer drug, daunorubicin [58]. The cholesterol-lowering drug, Atorvastatin, has also been reported to upregulate the expression of PPAR*δ* in Angiotensin II-induced hypertrophic cardiomyocytes *in vitro* [34]. Another study showed that digoxin could upregulate cardiac PPAR*δ* followed by improving lipid metabolism in the heart of diabetic rats [37]. Therefore, it appears that many currently used drugs may also exert PPAR*δ* activation effects, in addition to those well-designed and characterized PPAR*δ*-selective ligands. However, the underpinning mechanisms for their PPAR*δ* activation effects need further investigations.

Besides the endogenous and synthetic PPAR*δ* ligands, many naturally occurred compounds from plants have been shown to either act as PPAR*δ* ligands or PPAR*δ* enhancers. For example, green tea extract selectively activates PPAR*δ* in cultured cardiomyocytes [116, 117]. A recent study demonstrates that physiological levels of caffeine enhance cell metabolism at least partially via PPAR*δ* [118]. Although the *in vivo* effects of green tea and caffeine on the adult heart need further studies, the clinical implication in the tea and coffee drinking population is intriguing. Further study is required to determine if drinking tea or coffee may activate cardiac PPAR*δ* and may exert beneficial effects as those we shown in transgenic mice with heightening cardiomyocyte-restricted PPAR*δ* expression [40]. Baicalin is a natural flavone found in the extract of the rhizome of the perennial herb *Scutellaria baicalensis*, known as huangqin in China and is one of the most used herbs in Traditional Chinese Medicine for thousands of years [119]. Zhang et al. showed that baicalin restores cardiac PPAR*δ* expression *in vivo* and *in vitro* in cultured cardiomyocytes [35]. Abscistic acid, a phytohormone, is among those newly identified PPAR*δ* activators [120]. The cyclohexenyl chalcone panduratin A, isolated from *Boesenbergia pandurata* rhizomes, was shown to be a natural AMPK stimulator, with consequent activation of PPARα/*δ* [121]. Therapeutic potential of panduratin A, an LKB1-dependent AMP-activated protein kinase stimulator, is shown to activate PPARα/*δ* and can be an effective anti-obesity treatment [121]. Vaticanol C is reported to be a complex resveratrol tetramer activates PPARα and PPAR*δ*, but not PPAR*γ* both *in vitro* and *in vivo* [122]. Ginsenoside Rh2 treatment in type1-like diabetic rats can enhance cardiac expression of PPAR*δ* and repress cardiac fibrosis [123]. The list of new identified natural activators of PPAR is still expanding. However, it remains unclear how we may use these potential natural PPAR*δ* activators for prevention and alternative treatments of cardiac disorders. These studies do not provide potential mechanisms underlying the upregulation of cardiac PPAR*δ*. At present, studies are still absent to further determine which of the two PPAR subtypes (*α* and *δ*) exert the cardiac protective effects of these compounds. In systemic treatment, the cardiac PPAR*δ* activators could have been due to their effects on other systems, eg., PPAR*δ* exerts antihypertensive effects [124]. While the potential anti-hypertensive effect of activating PPAR*δ* post additional beneficial effect when treating heart failure patients with hypertension, other systemic effects of PPAR*δ* remain major confounding factors preventing the potential application. Therefore, it appears that the understanding of the molecular mechanisms for the specific changes in cardiac expression and activation of PPAR*δ* are surprisingly scarce. Further investigations in the regulation of cardiac PPAR*δ* may yield crucial insights into translatable therapies for treating heart disease.

## 5. Future Perspectives and Conclusion

As ligand-activated transcription factors, the PPARs represent an attractive target for the development of therapeutic agents. Synthetic PPAR*δ* agonists such as GW0742, GW501516, and L165041 [110–113] have been developed and even went on to early clinical trials. Although controversial, the main concern remains to be its potential tumorigenesis effects (see review [125]). The systemic use of PPAR*δ* is undoubtedly problematic because its seemingly differential effects on different cell types and tissues, which is evident by numerous studies using global and tissue-specific PPAR*δ* transgenic and gene-targeting mice [30, 41, 69, 126–128]. On-going efforts on designed-synthesized tissue-specific PPAR*δ*-selective ligands [129, 130] may give hope for targeting cardiac PPAR*δ* for the treatment of metabolic derangement and mitochondrial dysfunction in cardiac pathological states.

Despite the identification of numerous natural ligands for PPARs, a major challenge is the lack of in-depth studies on most of them. New approaches have been proposed using the cellular and biophysical pipeline for the screening of PPAR*δ*-selective ligands to avoid false positives [131]. Another alternative approach aims at further characterizing PPAR*δ* targets to delineate relevant downstream regulatory pathways to avoid the unwanted effects.

## Figures and Tables

**Figure 3:**
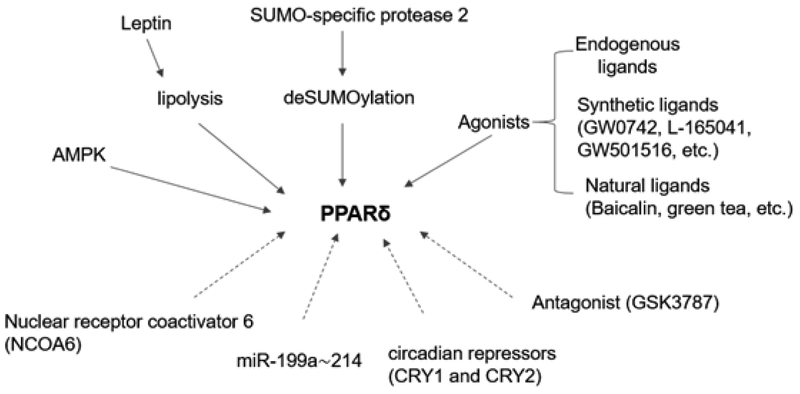
Upstream regulation of PPAR*δ* in the heart. The cardiac expression and activity of PPARδ can be elevated by various PPARδ agonists (endogenous, synthetic and natural ligands), post-translational modification (deSUMOylation), leptin and AMPK (AMP-activated protein kinase). In contrast, PPARδ antagonists, microRNA (miR-199a-214) and nuclear receptor coregulator (Nuclear receptor coactivator 6). Arrows with solid lines: activation; Arrows with dash lines: inhibition.
